# Comprehensive examination of tattoo removal using a 150 ps Nd:YAG laser in a porcine model

**DOI:** 10.1038/s41598-023-40379-z

**Published:** 2023-08-11

**Authors:** Justinas Baleisis, Romualdas Rudys

**Affiliations:** Department of Biomodels, State Research Institute Centre for Innovative Medicine, Santariskiu st. 5, 08410 Vilnius, Lithuania

**Keywords:** Biophysics, Medical research, Experimental models of disease, Outcomes research, Preclinical research, Translational research, Optics and photonics, Applied optics, Lasers, LEDs and light sources, Other photonics

## Abstract

This study aimed to investigate the efficacy of a Nd:YAG laser with a pulse duration of 150 ps at different laser parameters. The effects on multiple-colored tattoos with such ultrashort pulses has not been previously described in the literature. In vivo experiments were conducted on porcine skin to analyze the fragmentation efficiency of five different tattoo colors using different wavelengths, pulse energies, and spot sizes. The results showed that the optimal tattoo clearance to safety ratio for blue, green, red, and yellow tattoos with a 532 nm wavelength was 0.96–2.39 J/cm^2^. The laser with a wavelength of 1064 nm demonstrated the highest efficacy in eliminating black tattoos, with positive results observed for green and blue pigments at a fluence of 3.02 J/cm^2^. The study provides valuable insights into the efficacy of laser treatment with 150 ps for removing tattoos of different colors using different laser parameters. This information can help dermatologists and practitioners perform more efficient and effective tattoo removal with fewer side effects.

## Introduction

The cultural significance of tattoos has undergone a shift in recent years, with their increasing normalization in various forms of media. This has resulted in a greater availability of information regarding tattoo designs, artists, and hygiene precautions to the general public^1^. However, this has also led to a higher prevalence of "tattoo regret" among certain demographic groups, particularly among millennials, which is anticipated to drive the demand for tattoo removal methods in the coming years^2^.

Currently, laser technology is the most widely utilized method for tattoo removal and is expected to continue to account for the majority of revenue contributions in this market. The appeal of laser technology is attributed to a variety of factors, including increased public awareness, faster healing times, and a reduction in side effects compared to alternative methods. However, the complete removal of tattoos still poses a significant challenge, with factors such as tattoo complexity^3^, ink properties^4^, skin type, and age of tattoo all impacting the success of removal^5^. The potential for both short-term and long-term side effects associated with tattoo removal continue to be a significant concern. These may include temporary side effects such as pain, erythema, and infection, as well as long-term effects such as scarring and changes in pigmentation^6^.

For the past two decades, the mainstay of tattoo removal has been the utilization of Quality-switched (QS) lasers^7^. These lasers produce pulses with peak energies of up to 10 J/cm^2^ and pulse durations within the nanosecond range^8^. However, the pulse duration range of these lasers is still not sufficient to effectively break down ink particles while minimizing damage to surrounding tissue^9^. In recent years, picosecond (ps) lasers have emerged as a more promising option for tattoo removal, as they have been shown to be more effective in terms of tattoo clearance and pain management in both preclinical^10^ and clinical studies^11^.

This tattoo removal technique is based on the photoacoustic effect, which is characterized by the rapid thermal expansion of targeted tissue following fragmentation and the release of mechanical supersonic or acoustic waves, resulting in the destruction of surrounding tissue^12^. The resulting fragmentation of pigments into smaller fragments allows for their phagocytosis by macrophages and subsequent removal via the lymphatic system^13^, leading to tattoo lightening^14^. Additionally, endothermic steam carbon reactions may also alter the optical properties of tattoo inks, reducing their visibility^15^. At equal laser irradiance, a 150-ps laser pulse will result in 100 times greater tensile stress at the tattoo target than obtained using a 15-ns Q-switched laser pulse. This greater mechanical stress significantly improves the probability of fragmenting the tattoo particle and suggests that it may be possible to use pulses with lesser irradiance, without degradation in the outcome and possibly greater safety^16^.

As the laser pulse duration is crucial for pigment fragmentation^10^, the commercially available picosecond-domain laser systems mostly excel at 300–600 ps^17^. We aimed to investigate the laser-tissue interactions of an Nd:YAG laser with switchable wavelength (532/1064 nm) and spot size (2/4 mm) at various pulse energies (25–155 mJ), that can generate pulses of 150 ps. To date, there is a lack of published literature on the effectiveness and safety of 150 ps laser systems to remove tattoos, but in our previous study^18^, we evaluated the healing patterns of laser-induced microlesions on vivo porcine skin using microlens array optics. In this in vivo study, we conducted a comprehensive analysis of intradermal tattoo ink pigment fragmentation efficiency and treatment safety. We used a non-invasive multispectral imaging technique (SIAscopy) in combination with clinical evaluation, image processing software, and histopathological analysis. Our study was conducted on porcine skin, which closely resembles human skin in many regards.

## Materials and methods

### Ethics statement and animal model

The Lithuanian Laboratory Animal Use Ethical Committee under the State Food and Veterinary Service approved all procedures used in this study.

In this study, we employed a male Lithuanian White pig (Sus scrofa domestica) aged 12 weeks (n − 1) and weighing 50 kg to create multiple tattoos that were subsequently removed using laser procedures. The Department of Biomodels at the State Research Institute Centre for Innovative Medicine (Vilnius, Lithuania) held the animal, and all actions carried out adhered to the ARRIVE guidelines and the Directive 2010/63/EU of the European Parliament and the European Council on the protection of animals used for scientific purposes. The experiment was conducted in accordance with institutional standard operating procedures, environmental enrichment and veterinary care were provided. The animal holding areas were maintained at a temperature of 20 to 24 °C, with a 12:12 h light–dark cycle and a humidity of 40 to 60%. Access to fresh water was provided ad libitum and feed was supplied three times a day.

### Experimental design

**Figure 1:**
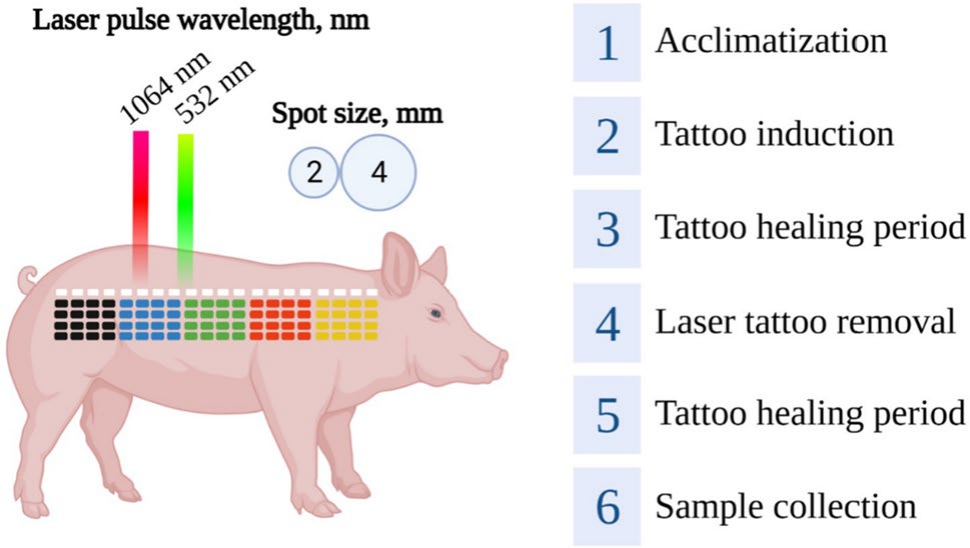
Experimental design outlining the procedure of tattoo application, followed by a healing phase, subsequent laser treatment, and final sample collection.

The study was designed to encompass a pre-experimental acclimation period of 4 weeks, followed by the initiation of tattooing procedures on day 0. The tattoo healing phase was then observed over a period of 30 days. Three laser treatments in all were carried out, with a 30-day recovery period in between each. On day 150, sample collection was conducted and upon completion, the animals were humanely sacrificed to obtain skin samples for further analysis. The study design is illustrated in Fig. 1.

The animals were euthanized using a sodium pentobarbital-based solution (Exagon, Richter Pharma AG, Austria) at a dose of 50 mg/kg, following sedation with ketamine hydrochloride (Ketamidor, Richter Pharma AG, Austria) at a dose of 15–20 mg/kg and xylazine hydrochloride (Sedaxylan, Eurovet Animal Health B.V., Netherlands) at a dose of 2 mg/kg. Euthanasia was performed in accordance with established protocols for humane end-of-life treatment.

### Preparation of tattoo model

Anesthetized animals were prepared for tattooing utilizing a 3–4% concentration of isoflurane (Isoflurin 1000 mg/g, Vetpharma animal health, S.L., Spain) administered at a flow rate of 4–5 L/min. Prior to tattooing, the animals were positioned in the prone position and the hair on the dorsolateral thoracic and lumbar regions was clipped using an electric clipper (Golden A5 5-50, Oster, Switzerland). The designated tattoo areas were cleaned with a solution of warm water and 70% ethanol (Art. No. P075.1, Carl Roth GmbH & Co KG, Germany).

We selected a range of commonly used, widely available and currently authorized in Europe inks of five different colors, each with an identified composition as outlined in Table 1.

**Table 1:** Tattoo ink composition.

Ink color	Black	Blue	Green	Red	Yellow
Composition of tattoo inks	C.I. 77266 (paracrystalline carbon)	C.I. 74160 (copper phthalocyanine; C_32_H_16_CuN_8_)	C.I. 11740 (pigment yellow 65; C_18_H_18_N_4_O_6_) C.I. 77891 (titanium dioxide; TiO_2_) C.I.74100 (pigment blue 16; C_32_H_18_N_8_)	C.I. 56110 (organic pigment red 254, C_18_H_10_Cl_2_N_2_O_2_) C.I. 11740 (pigment yellow 65; C_18_H_18_N_4_O_6_) C.I. 21110 (pigment orange 13; C_32_H_24_C_l2_N_8_O)	C.I. 11740 (pigment yellow 65; C_18_H_18_N_4_O_6_) C.I.7789 (titanium dioxide; TiO_2_) C.I. 77120 (barium sulfate, BaO_4_S)

Color index constitution number (C.I.) of pigments provided by the tattoo ink manufacturers.

The tattoos were applied using a raster technique (straight lines back and forth) covering the entire area, with missing areas being filled by repeating the process. A rotary tattoo machine was utilized to inject the tattoo ink into the skin at a depth of 2 mm. The distance between each tattoo and the next one was 0.5 cm, and each tattoo was 2 cm by 1 cm in size. A total of 16 areas per ink color was applied.

### Laser procedure

A neodymium-doped yttrium aluminum garnet (Nd:YAG) picosecond laser system (PicoClarans, Photosana, Lithuania) with a zoom handpiece was utilized for laser tattoo removal. The system employed a pulse duration of 150 ps and a switchable wavelength of 532 and 1064 nm [stimulated Brillouin scattering (SBS) compressed Q-switched laser]. In this study, 2 mm and 4 mm spot sizes were selected. A power/energy meter (LabMax-Top, Coherent, USA) was utilized to calibrate the laser system prior to each procedure, to ensure uniform pulse generation. During the laser treatment, the handpiece was positioned perpendicular to the intended skin areas and moved back and forth in a zigzag pattern. Each laser pulse was delivered at a rate of 4 Hz until the entire area was covered with an appropriate laser setting, as specified in Table 2.

**Table 2:** Energy density parameters of the Nd: YAG laser system used in the study.

Wavelength		532			1064		
Pulse energy, mJ		25	75	120	30	95	155
		Fluency, J/cm^2^					
Spot size, mm	2	0.80	2.39	3.82	0.96	3.02	7.96
	4	0.20	0.60	0.96	0.24	0.76	1.99

### Sample preparation

Samples were collected 30 days following the third laser procedure. To prevent cross-treatment contamination, 6 mm wide punch biopsy needles (33–36–6 mm, Integra LifeSciences, USA) were used to collect biopsies from the center of each treated area. The collected skin biopsies were fixed in 10% formalin (Art. No. A146.5, Carl Roth GmbH & Co KG, Germany) and embedded in paraffin blocks (with 1 block containing 1 skin biopsy). The paraffin blocks were then cut into 4um sections using a rotary microtome (RM2255, Leica Microsystems IR GmbH, Germany). The sections were stained using hematoxylin (Art. No. T864, Carl Roth GmbH + Co KG, Germany) and eosin Y (Art. No. 3137.1, Carl Roth GmbH + Co KG, Germany) according to the manufacturer's recommended protocol. The stained tissue slides were scanned using a brightfield digital pathology scanner (Aperio ScanScope XT, Leica Microsystems IR GmbH, Germany), and the data was processed using ImageJ software (ver. 1.53r, National Institutes of Health, Bethesda, MD, USA).

### Tattoo clearance analysis

SIAscopy a non-invasive multispectral imaging technique (SIMSYS-MoleMate, MedX Health, USA), was utilized to capture dermatoscopic photographs of tattoo clearing dynamics. These images were then analyzed using an image processing program. The following steps were taken to extract color channels from the examined dermatoscopic images: selecting the Image → Type → RGB stack option. The most appropriate color channel was selected based on the tattoo pigments as follows: blue channel for yellow pigments, red channel for black, blue, and green pigments, and green channel for red pigments. Using Image → Adjust → Threshold option with the Stack histogram option, the pigment was marked by specifying limits for pixel intensity. The marked areas were then measured using the "Measure" function. Each area was evaluated over a total surface area of 0.95 cm^2^. The evaluation was performed prior to sample collection, with the device placed at the center of each area.

### Laser induced skin irritation score

The safety of a picosecond-domain laser system was evaluated at various settings by assessing the skin irritability that occurred after laser treatment, using the standard for biological evaluation of medical devices (ISO 10993-23:2021, Chapter 7). The degree of erythema and edema was used to assign the following scores based on severity: 0 for no perceptible changes, 1 for barely perceptible changes, 2 for well-defined changes, 3 for moderate changes, and 4 for severe changes. The total score (with a maximum of 8 points) was calculated by summing the individual evaluations of each characteristic (Fig. 4).

### Statistical analysis

Mann–Whitney U test (abnormal distributed variables) was used to compare the laser induced skin irritability between spot sizes of the same pulse energy. The threshold for statistical significance was set at P < 0.05. All calculations were made using Origin (Pro), Version 9.9 2022 (OriginLab Corp., USA).

### Institutional review board statement

All procedures performed in this study were approved by the Lithuanian Laboratory Animal Use Ethical Committee under the State Food and Veterinary Service.

## Results

### Macroscopic laser-tissue irritation evaluation

**Figure 2:**
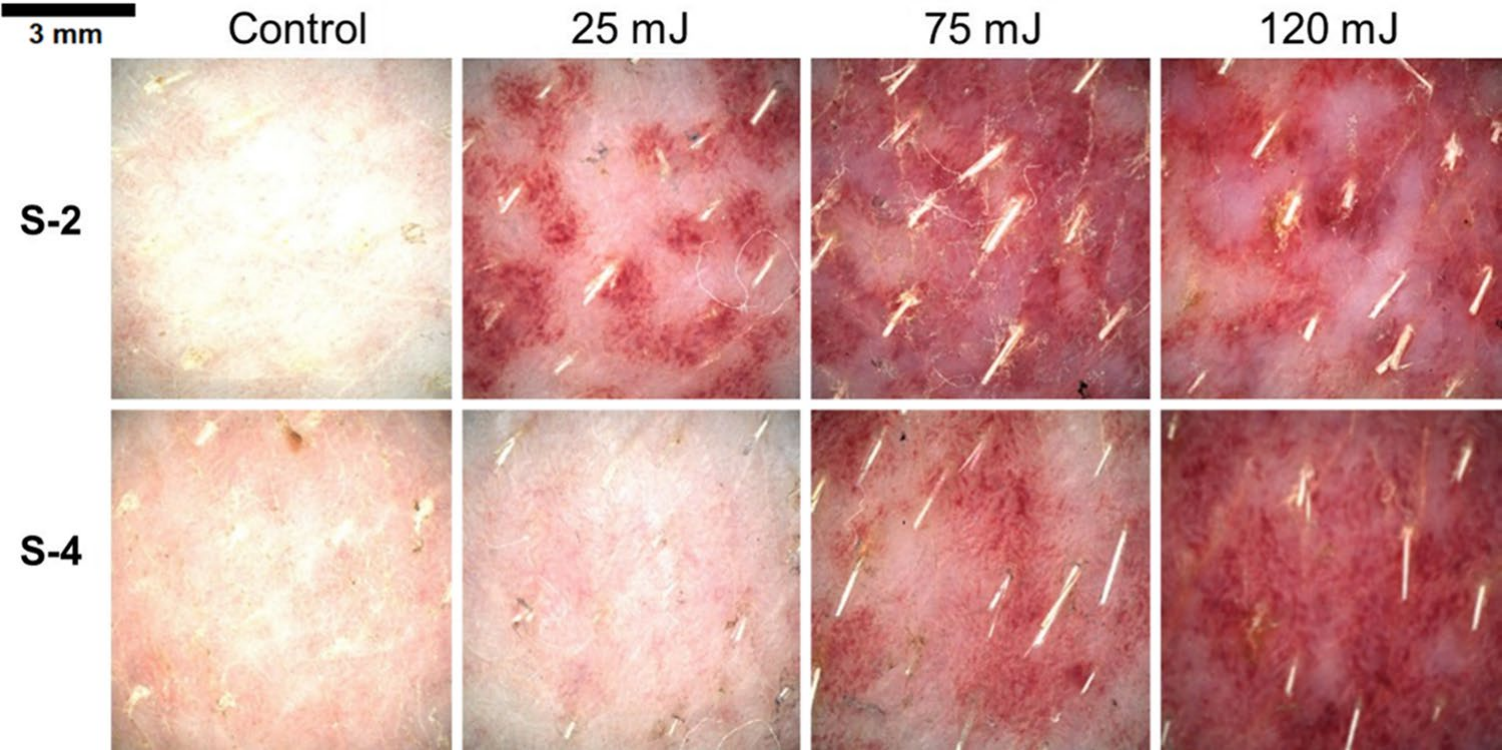
Dermatoscopic evaluation of untattooed skin immediately following treatment with 532 nm laser therapy.

**Figure 3:**
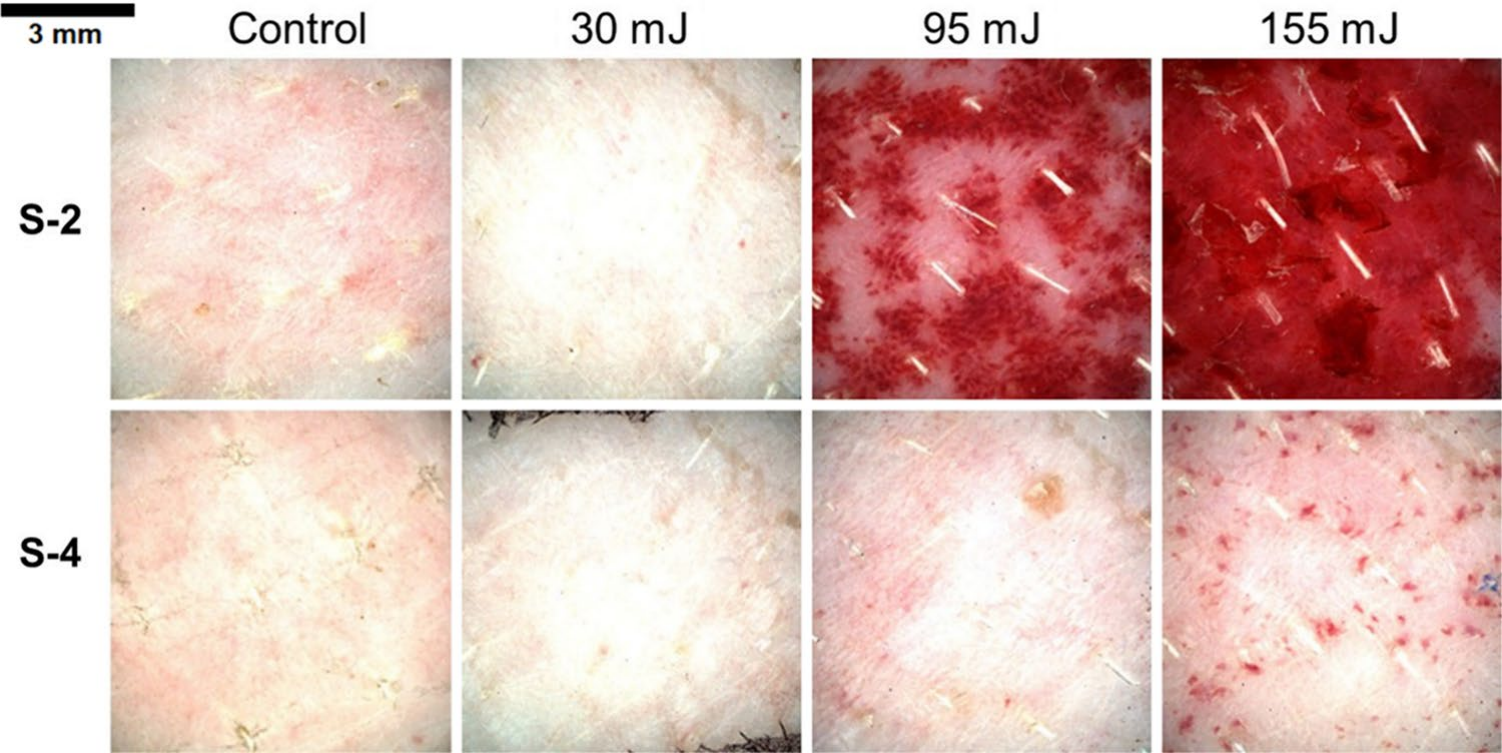
Dermatoscopic evaluation of untattooed skin immediately following treatment with 1064 nm laser therapy.

**Figure 4:**
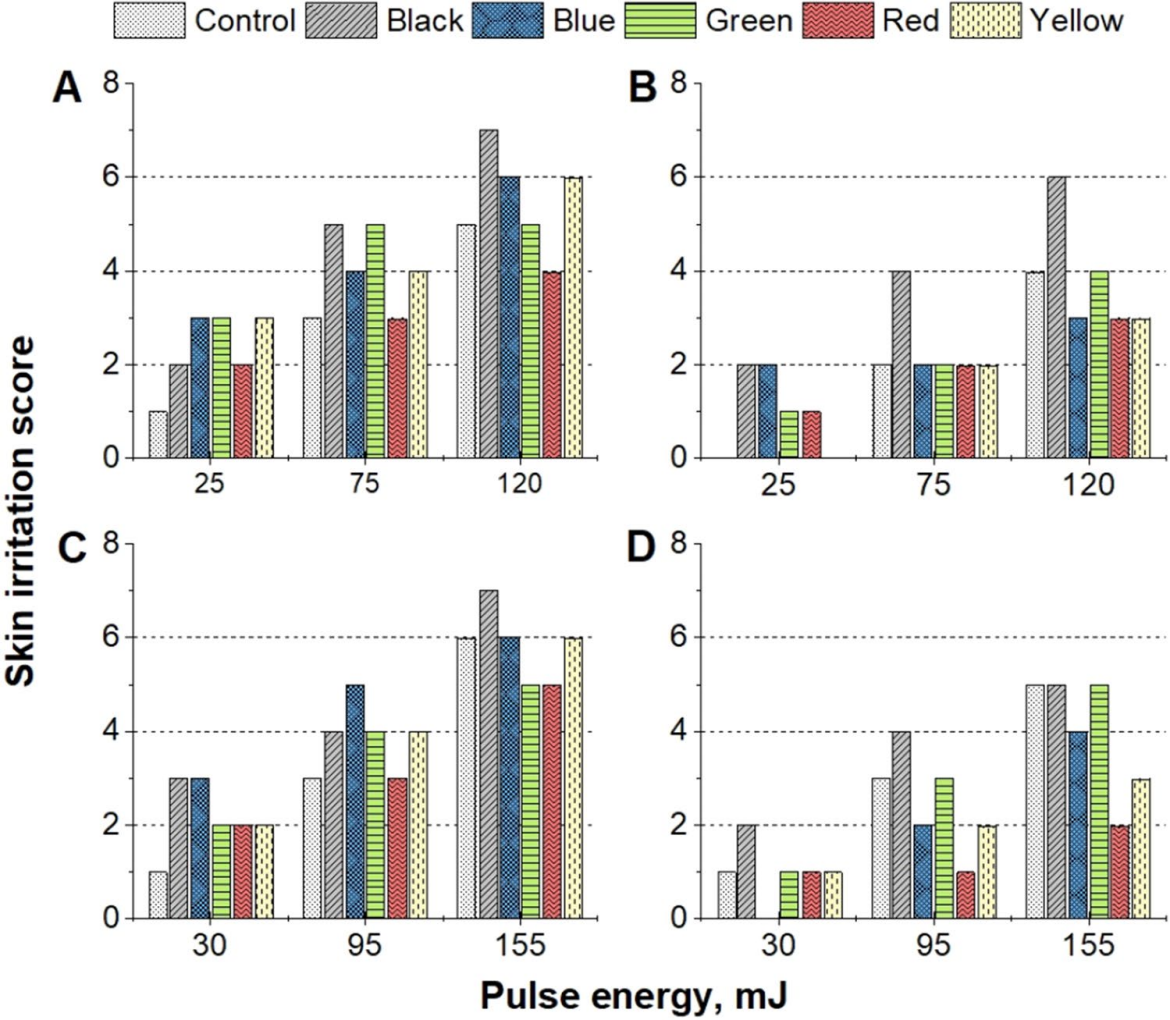
Assessment of tissue irritation immediate post-laser treatment. Laser treatment parameters: (**A**) wavelength of 532 nm and 2 mm spot size; (**B**) wavelength of 532 nm and 4 mm spot size; (**C**) wavelength of 1064 nm and 2 mm spot size; (**D**) wavelength of 1064 nm and 4 mm spot size.

For establishing a reference for skin irritation, healthy skin was exposed to the same laser parameters as the laser-treated areas (Figs. 2 and 3). Areas irradiated with 532 nm wavelength were more severe using the 2 mm spot size beam, that produced intense and localized erythema. Barely perceptible edema increased in severity past 75 mJ pulse energy and became moderate at 120 mJ (Fig. 2S-2). In comparison, the irradiation effect with 4 mm spot size was milder, resulting in a well-defined erythema and edema only at 120 mJ, and the erythema that was developed by the pulse energy of 75 and 120 mJ had a uniform distribution (Fig. 2S-4).

The laser treatment on healthy skin using the 1064 nm wavelength displayed almost no indications of laser-induced patterns at 30 mJ, but a rapid increase in erythema was observed past 95 mJ with the 2 mm spot size, developing a severe form (Fig. 2S-2). Areas treated with the 4 mm spot size up to 95 mJ pulse energy were comparable to healthy skin. Sporadic petechia formation (< 1 mm) was observed in areas treated with 155 mJ using the 4 mm spot size (Fig. 2S-4).

The degree of tissue irritability was assessed to ascertain how various laser parameters affected different colored tattoos. The erythema and edema for all areas were overall reduced (Fig. 4B) using a 4 mm spot size at a 532 nm wavelength in comparison to the treatment using a 2 mm spot size (p = 0.054) (Fig. 4A). The severity of tattoo removal, as measured by the outcome of treatments utilizing 75 mJ (p = 0.011) and 120 mJ (p = 0.041) energy levels and matching pigment color, was found to be higher when using a 2 mm spot size compared to a 4 mm spot size.

When utilizing a 2 mm spot size, pulse energies of 75 to 120 mJ, and a 532 nm wavelength, black tattoos exhibited the most pronounced response to laser treatment, resulting in moderate to severe edema (Fig. 4A). The laser-tissue reactions elicited by 532 nm wavelength were found to be the mildest in red and green tattoos, with only moderate edema observed even at the highest pulse energy levels. The analysis of the data presented in Fig. 4B revealed that, apart from black tattoos, all laser-treated areas subjected to irradiation with 120 mJ pulse energy using a 4 mm spot size resulted in comparable or reduced irritation scores when compared to the control skin.

The skin irritation scores of the areas treated with 1064 nm wavelength were significantly higher (p – 0.037) after treatment with 2 mm spot size compared to 4 mm spot size (Fig. 3C, D). The use of a 2 mm spot size at both 30 mJ and 155 mJ pulse energies resulted in a significantly higher irritation score compared to the 4 mm spot size. Specifically, at 30 mJ pulse energy, the 2 mm spot size resulted in a 2.7-fold increase in irritation score, primarily due to the presence of edema. Similarly, at the highest pulse energy level of 155 mJ, the use of a 2 mm spot size led to a 1.9-fold higher irritation score (p = 0.013) compared to the 4 mm spot size, as shown in Fig. 3D.

The overall irritability of the treated areas was found to be highest in black, blue, and yellow tattoos, due to lower edema severity at pulse energies ranging from 30 to 95 mJ with a 2 mm spot size (Fig. 4). Conversely, the treatment of red and green tattoos resulted in the least irritation compared to the other colors, due to a slight reduction in edema formation.

### Tattoo clearance evaluation

**Figure 5:**
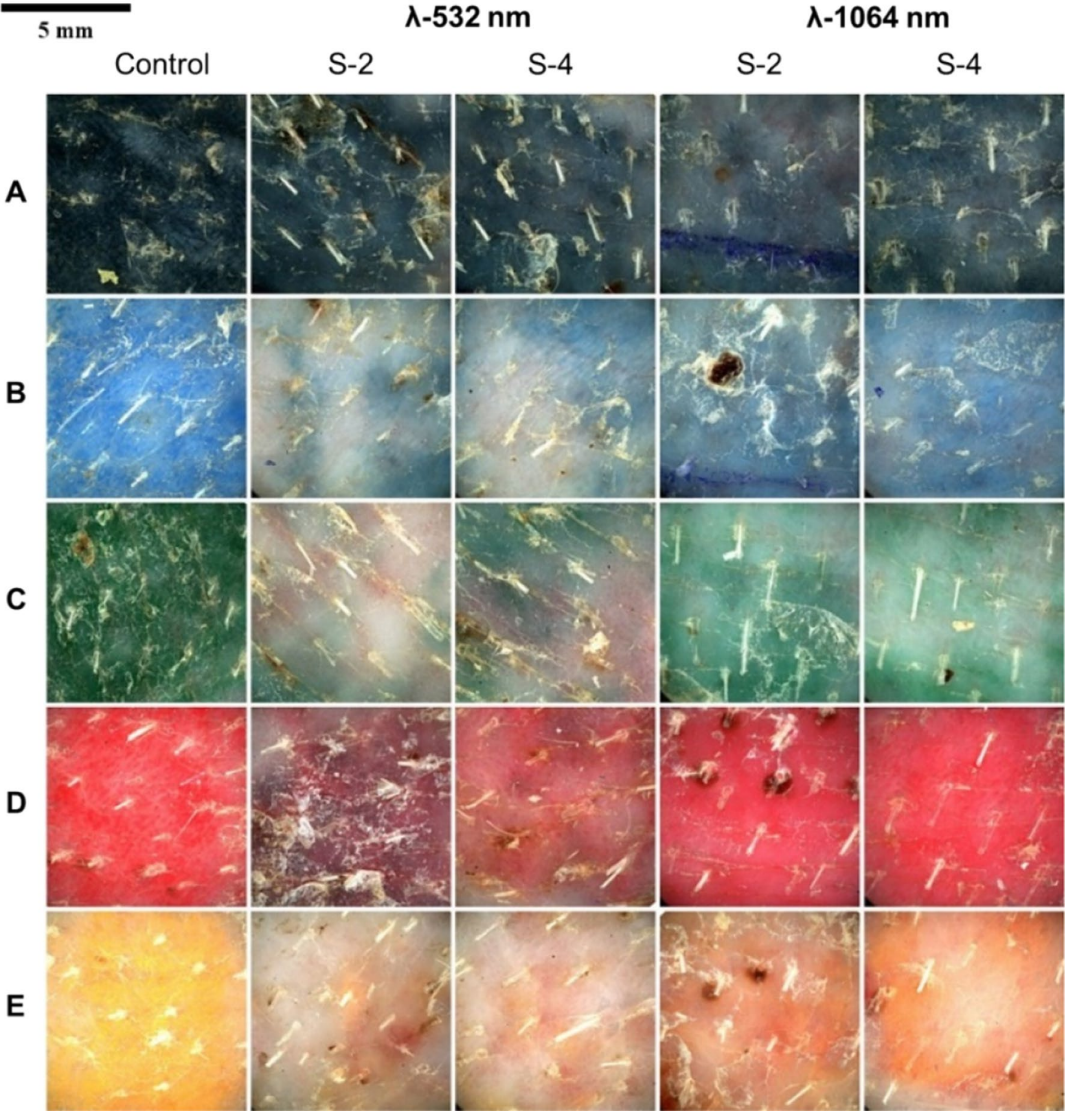
Dermatoscopic view of laser-treated tattoos 30 days post third treatment. For 532 nm wavelength a pulse energy of 75 mJ was used and for 1064 nm – 95 mJ. Tattoo color—(**A–E**). Spot size (S)—2- and 4-mm. Treatment wavelength (λ)—532/1064 nm.

Different wavelengths of laser light have varying degrees of effectiveness in tattoo removal. The clearance rates demonstrated a positive correlation with pulse energy. When comparing the clearance rates achieved using different wavelengths, it is observed that the 1064 nm wavelength produced lower clearance rates compared to the 532 nm wavelength, with the exception of black tattoo (Fig. 5).

**Figure 6:**
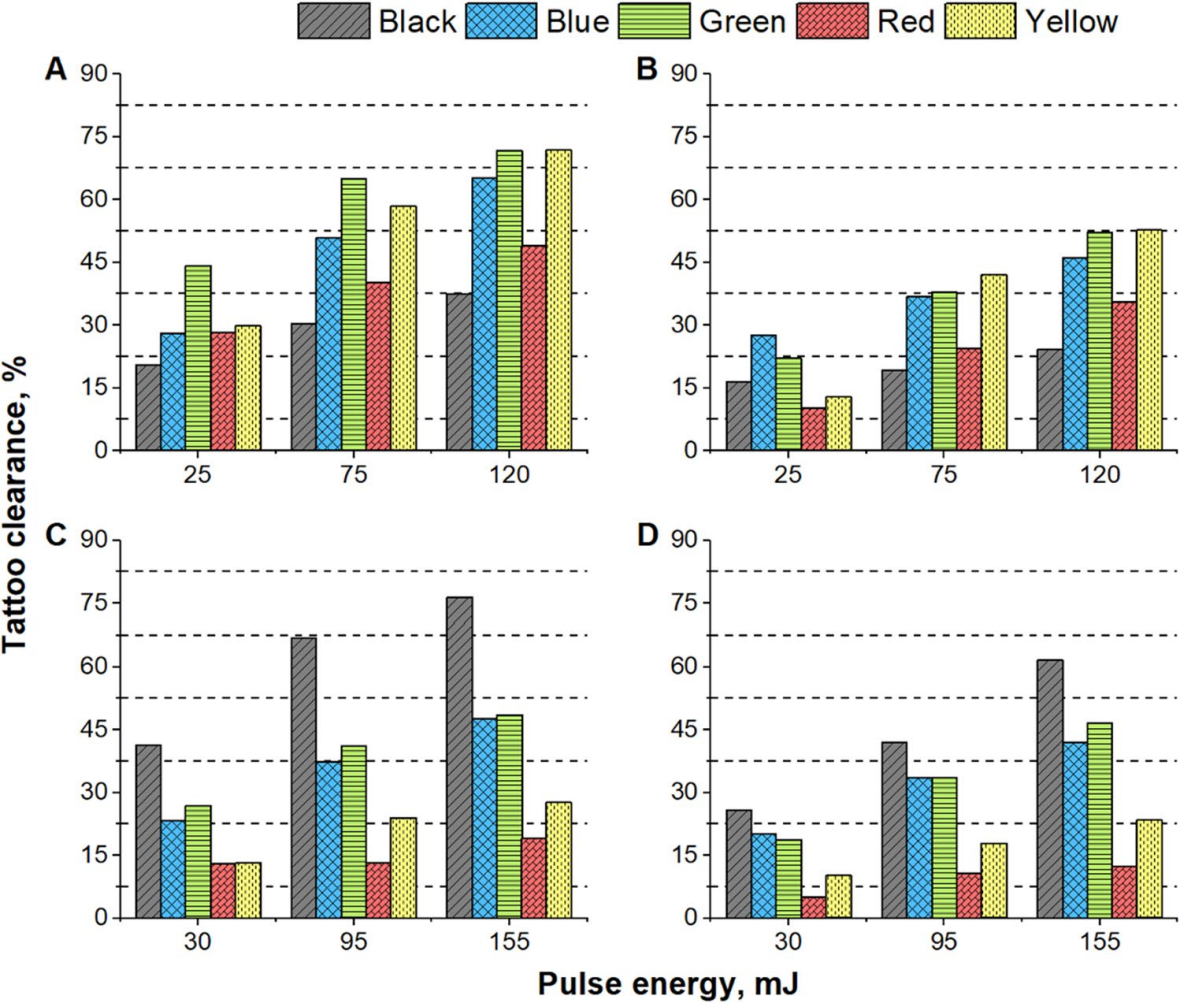
Assessment of tattoo clearance 150 days post-treatment. Laser treatment parameters: (**A**) wavelength of 532 nm and 2 mm spot size; (**B**) wavelength of 532 nm and 4 mm spot size; (**C**) wavelength of 1064 nm and 2 mm spot size; (**D**) wavelength of 1064 nm and 4 mm spot size.

The highest clearance rate for black tattoos was achieved using a 2 mm spot size at 1064 nm wavelength and 155 mJ pulse energy, resulting in a rate of 76.46% (Fig. 6C). Conversely, the least effective treatment, which only achieved a clearance rate of 16.41%, was observed when using a 4 mm spot size at 532 nm wavelength and 25 mJ pulse energy (Fig. 6B).

The most effective treatment for removing blue, green, red, and yellow tattoos utilized a beam size of 2 mm, a wavelength of 532 nm, and an energy of 120 mJ (Fig. 6A). After three laser treatments, yellow and green tattoos exhibited the highest clearance rates, achieving 71.87% and 71.69%, respectively. Blue tattoos were the third most effectively treated, with a clearance rate of 65.04%, followed by red color tattoos, with a rate of 48.96%. The lowest clearance for blue, green, red, and yellow color tattoos was observed after treatment utilizing the 4 mm spot size at 1064 nm wavelength and 30 mJ pulse energy, achieving 20.24%, 18.74%, 5.15%, and 10.20% respectively (Fig. 6D).

### Microscopic tattoo removal evaluation

The histological characteristics of the epidermis in untreated and laser-treated tattoo samples were evaluated. The epidermis maintained its characteristic architecture after treatment with various laser settings. Furthermore, no extracellular ink particles were detected in the epidermis, even in the accumulations of necrotic tissue that formed in the stratum corneum of the epidermis.

**Figure 7:**
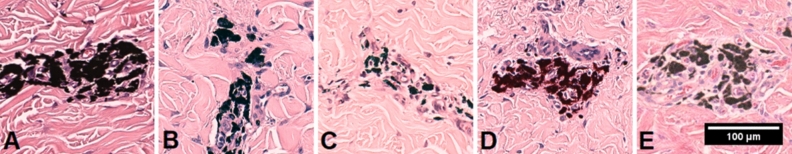
Histological analysis of pre-treatment tattoo pigments: control samples 150 days after tattooing. HE-stained sections of pigment clusters in different colored tattoos: (**A**) black tattoos; (**B**) blue tattoos; (**C**) green tattoos; (**D**) red tattoos; (**E**) yellow tattoos.

The skin tissue slides stained with hematoxylin and eosin (HE) showed variations in ink color of non-laser treated areas, with black tattoos appearing black, blue tattoos displaying a range from dark blue to black with a bluish border, green tattoos exhibiting a range from dark green to black with a greenish border, red tattoos showing a range from dark red to black with a reddish border, and yellow tattoos presenting a heterogeneous black color with multiple pigment granules (Fig. 7).

**Figure 8:**
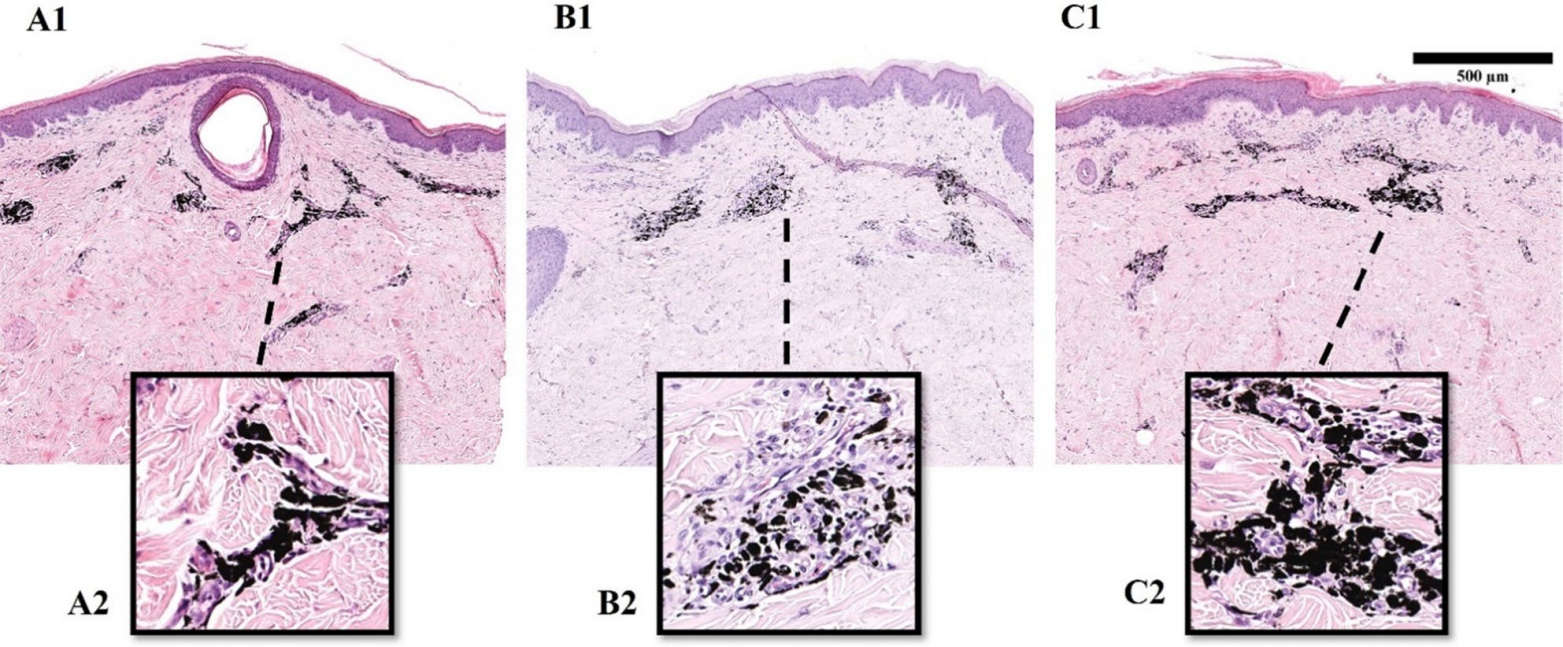
Histological assessment of black tattoos 150 days post-laser treatment with 532 nm wavelength and 120 mJ pulse energy: (**A1**) control (untreated) tattoo; (**A2**) black pigment cluster in untreated tattoo; (**B1**) laser-treated tattoo (2 mm spot size); (**B2**) pigment particles in laser-treated tattoo (2 mm spot size); (**C1**) laser-treated tattoo (4 mm spot size); (**C2**) pigment particles in laser-treated tattoo (4 mm spot size).

Ink particles were observed in the dermis up to a depth of 1.2 mm, with the highest concentration locating within 100—600 μm from the skin surface (Fig. 8A1–2). The majority was concentrated in clusters distributed in mononuclear infiltration, comprising mainly of macrophages surrounding dilatated blood vessels (Fig. 8). The cytoplasm of phagocytic cells contained large pigment deposits, which showed a high degree of color saturation and homogeneity, except for yellow tattoos.

The study showed that 532 nm wavelength at the 4 mm spot size only reached up to 250 μm (Fig. 8B1–2), while the 2 mm spot size was capable of achieving pigment fragmentation up to 400 μm (Fig. 8C1–2). Reduction in overall pigment quantity and the transformation of large intracellular pigment deposits into considerably smaller particles containing granules resulted in a lightening of the treated areas. By increasing the laser pulse energy from 75 to 120 mJ, the fragmentation effect was significantly enhanced, as demonstrated by the tattoos' clearance (Figs. 5 and 6**)**.

Laser treatment at 532 nm wavelength produced detached melanin‐containing parakeratotic mounds or microscopic epidermal necrotic debris (MEND) that reside in the upper levels of the SC in sub-granular position. The laser beam spot size, pulse energy, and pigment characteristics affected the size variation of MENDs. The treatment with a 2 mm spot size resulted in the formation of MENDs in blue, green, and yellow tattoos, especially past 75 mJ pulse energy. The largest MENDs were observed in yellow tattoos, measuring up to 1.5 mm in diameter. In blue tattoos, the detached necrotic tissue was measured up to 500 μm, and in green tattoos—up to 300 μm. MENDs were not observed in black and red tattoos, and they were not observed in tattoos of any color treated with a 4 mm spot size. Vascular dilatation of superficial capillaries was observed throughout the samples treated with 532 nm wavelength and both spot sizes. Using the 2 mm spot size the dilatation was observed up to 1.5 mm in the dermis and using the 4 mm—up to 500 μm (Fig. 8).

**Figure 9:**
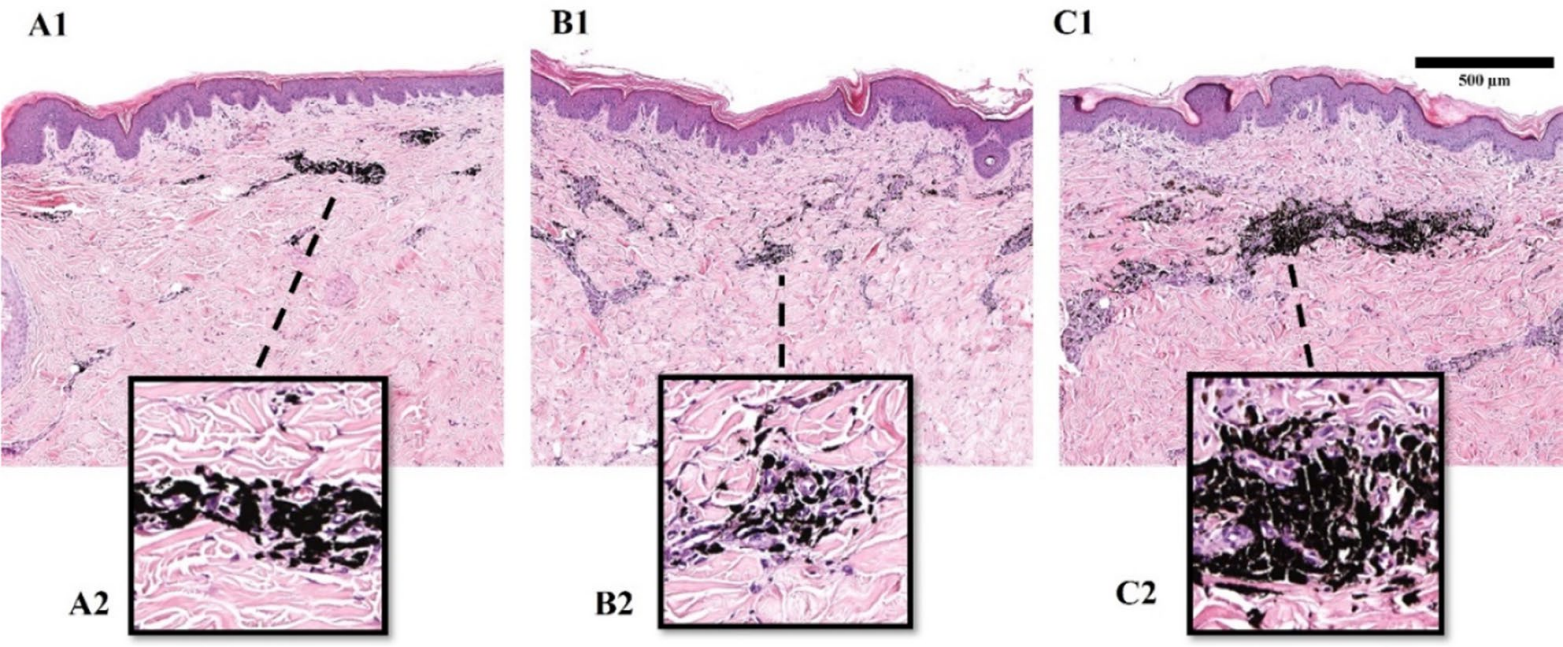
Histological assessment of black tattoos 150 days post-laser treatment with 1064 nm wavelength and 155 mJ pulse energy: (**A1**) control (untreated) tattoo; (**A2**) black pigment cluster in untreated tattoo; (**B1**) laser-treated tattoo (2 mm spot size); (**B2**) pigment particles in laser-treated tattoo (2 mm spot size); (**C1**) laser-treated tattoo (4 mm spot size); (**C2**) pigment particles in laser-treated tattoo (4 mm spot size).

The application of a 1064 nm wavelength for tattoo removal was particularly effective in decreasing the amount of pigment deposits in black tattoos (Fig. 5), especially when a 2 mm spot size was used (Fig. 9B1). The total black pigment amount in the samples treated with both beam sizes were lower than in the untreated areas (Fig. 9A1–2), which positively correlated with pulse energy past 95 mJ using a 4 mm spots size and 30 mJ with 2 mm spot. Treatment up to 95 mJ with the 1064 nm laser using a 4 mm spot size affected mostly the superficial layer of the black pigment clusters up to 300 μm, leaving the lower layers visually comparable to control tattoos (Fig. 9C1–2). Fragmentation of pigments throughout their distribution depth was observed to be successful after irradiation with a 2 mm beam width at all energy levels, and with a 4 mm beam width from 155 mJ pulse energy (Fig. 9B1–2).

Our observations indicate that colored tattoos exhibited fragmentation to some degree throughout the entire range of pigment distribution. In addition, we noted a slight dilatation of superficial capillaries up to 300 μm, along with a significant infiltration of mononuclear cells in all areas containing pigment. MENDs were only observed in histological samples of blue, green, and yellow tattoos treated with 2 mm spot size. The highest MEND formation was observed in blue and yellow tattoos up to 1.5 mm in diameter past 95 mJ, while in green tattoos the detached necrotic tissue measured up to 200 μm.

## Discussion

Picosecond laser tattoo removal works by delivering ultra-short pulses of high-intensity light energy into the skin^14^. This light energy is absorbed by the tattoo ink particles, causing them to break up into smaller pieces that can be naturally eliminated by the body's immune system^13^. The main mechanisms of picosecond laser tattoo removal include: photothermal, photomechanical and photoacoustic effect^12,15,19^. The shorter the pulse duration, the more precise and targeted the laser energy can be, allowing it to specifically target the ink particles without heating up the surrounding tissue and avoiding side effects^20^.

The effectiveness of laser treatment in tattoo removal depends heavily on the wavelength of the laser used^17^, as different tattoo colors have unique absorbance coefficients for specific wavelengths^4,21^. Hence, selecting the appropriate wavelength is crucial to maximize the clearance rate of the tattoo^1^. Our research shows that high-energy laser treatment is the most effective in removing tattoos, but it can also result in acute skin lesions like erythema, edema, and hemorrhage^6^. To balance the need for efficient tattoo removal with the risk of adverse events, the optimal ratio between safety and effectiveness must be evaluated. Our findings suggest that the optimal ratio varies based on fluence, pigment color and the applied wavelength. For instance, the optimal fluence range for removing blue, green, red, and yellow tattoos with 532 nm wavelength was 0.96–2.39 J/cm^2^. The 1064 nm laser was the most effective for removing black tattoos and showed some efficacy for removing green and blue pigments, achieving promising outcomes with a fluence of around 3 J/cm^2^. These results complemented other studies^3^, were the safest and most effective method for treating red and yellow pigments was with a 0.8 J/cm^2^ fluence at 532 nm wavelength. However, the tattoo removal procedures were conducted at longer pulse duration, than in our study, with 375 ps and 450 ps at a wavelength of 532 nm and 1064 nm, respectively.

The observed atypical response of green tattoos to green light in our study implies a more complex and nuanced interaction, that may be attributed to several factors. The specific green tattoo ink used in our study may possess broader absorption spectra, due to the presence of several pigments in its composition and can therefore absorb light outside the typical green wavelength. Furthermore, the utilization of a picosecond laser with a pulse duration of 150 ps may have enhanced the efficiency of the photomechanical impact, leading to effective pigment shattering. This advantageous effect was observed regardless of the precise match between the laser light and pigment absorption spectrum. Additionally, factors like tattoo depth, ink particle size, and the surrounding biological environment can also influence the overall absorption and scattering of light, potentially contributing to the observed response^19^.

The fading of tattoos following picosecond laser treatment can be attributed to two main factors: either a reduction in the quantity and size of tattoo pigments or their transformation into microscopic structures that cannot be detected optically^22^. A histopathological examination of laser-treated tattoos exhibited a decrease in the overall quantity of pigment present in the dermis. The breakdown of pigment was observed through changes in the intracellular accumulation of ink particles, which became heterogeneous in comparison to untreated tattoos. Furthermore, it was observed that using appropriate laser settings can induce a structural modification in the cytoplasm of macrophages containing intracellular pigment accumulations.

The fragmentation of pigment aggregates varied in depth, influenced by the absorption characteristics of the pigment particles at specific wavelengths and the intensity of the laser employed during treatment. Our findings demonstrate that by optimizing the treatment laser settings can influence the depth of pigment aggregate fragmentation at appropriate wavelength. Increasing the spot size and pulse energy of the 532 nm laser enables deeper penetration into the skin^23^, thereby enhancing the targeting of deeper tattoo pigments^24^. Notably, even when utilizing a consistent pulse energy of 120 mJ, we observed differences in the depths of tattoo aggregate fragmentation. Specifically, employing a 4 mm spot size resulted in breakdown reaching depths of up to 250 μm, whereas employing a smaller 2 mm spot size extended the fragmentation depth to 400 μm. It is noteworthy that despite the four-fold difference in fluence between the two spot sizes, the variation in penetration depth was not as substantial. These findings suggest that spot size plays a significant role in determining the depth of fragmentation, potentially due to the spatial distribution of laser energy.

When treating black ink tattoos at fluences up to 0.76 J/cm^2^ with a 1064 nm wavelength, we observed that pigment fragmentation occurred mainly in the upper layer of ink deposits, likely due to high absorption of the pigment. Reaching deeper pigment deposits would require more treatment sessions or higher fluencies, what was achieved by increasing fluencies from 1.99 to 7.96 J/cm^2^. To improve the penetration of the 1064 nm wavelength laser is the use of a large spot size or the R0 method. The R0 method involves using a low fluence to target the top layer of the tattoo pigment, followed by a high fluence to target deeper layers of the pigment^25^. In the histological samples of other tattoo colors, the pigment fragmentation was visible in all layers of the dermis, where its intercellular accumulations were detected. Thus, the absorption of pigments color does not alter the penetration depth of 1064 nm radiation, and higher fluence can increase the effectiveness of pigment fragmentation.

The results of the histopathological analysis demonstrate that laser treatment has the potential to generate microscopic epidermal necrotic debris (MEND) in the stratum corneum of the epidermis. The formation of MEND is dependent on various factors such as fluence and pigment properties, and its occurrence may lead to unfavorable skin reactions such as blistering, scabbing, scarring, and inflammation^26,27^. Furthermore, the formation of MEND may also impede the efficacy of the tattoo removal process by blocking the laser energy's penetration into the deeper layers of the skin, thus limiting the extent of pigment fragmentation and clearance^28^. Interestingly, we observed that 532 nm wavelength therapy resulted in the formation of MEND in all tattoo colors, with fluences ranging from 0.80 to 3.82 J/cm^2^, while 1064 nm irradiation only induced MENDs in blue, green, and yellow tattoos with fluences greater than 3.02 J/cm^2^. Understanding the factors that contribute to MEND formation is crucial because it can lead to adverse skin reactions^29^, which can affect patient comfort and recovery by impacting the degree of pigment fragmentation and clearance rate^30^.

## Conclusions

In conclusion, our study provides valuable insights into the effectiveness of a 150 ps Nd:YAG laser treatment for removing fresh tattoos of different colors. The use of a 4 mm spot size with both 532 nm and 1064 nm wavelengths induced a milder tissue reaction compared to treatment with a 2 mm spot size. Our findings also demonstrated a positive correlation between fluence used for treatment and tattoo clearance, as well as the severity of side effects. We identified an optimal fluence range of 0.96–2.39 J/cm^2^ for removing blue, green, red, and yellow tattoos with the 532 nm wavelength, and 3.02 J/cm^2^ for the effective removal of black tattoos using the 1064 nm laser.

Furthermore, our histological analysis showed that the laser treatment induces fragmentation of pigment deposits in macrophages, leading to a reduction in the overall tattoo pigment density in the dermis. However, individual factors such as ink composition, tattoo depth, and skin characteristics may impact clearance rates, and as such, our results may not be applicable to all tattoo types. Overall, our study highlights the potential of the 150 ps Nd:YAG laser treatment as an effective option for tattoo removal and provides insights into the optimal parameters for achieving successful outcomes.

## Data Availability

The data presented in this study are available on fair request from the corresponding author.
